# The Impact of Okra (*Abelmoschus esculentus*) Supplementation on Diabetes and Obesity Biomarkers in Type 2 Diabetes Patients: A Systematic Review and Meta‐Analysis of Randomized Controlled Trials

**DOI:** 10.1002/ptr.70071

**Published:** 2025-08-27

**Authors:** Ying Fan, Lei Wu, Yalan Zhang, Qin Hu, Jing Mei, Kousalya Prabahar, Benjamin Hernández‐Wolters, Hamed Kord‐Varkaneh, Changjiang Lei, Su Zheng

**Affiliations:** ^1^ Department of Cardiovascular Internal Medicine The Fifth Hospital of Wuhan Wuhan Hubei China; ^2^ Department of Orthopedics The Fifth Hospital of Wuhan Wuhan Hubei China; ^3^ Department of Laboratory Medicine The Fifth Hospital of Wuhan Wuhan Hubei China; ^4^ Department of Neurology The Fifth Hospital of Wuhan Wuhan Hubei China; ^5^ Department of Ultrasound Imaging The Fifth Hospital of Wuhan Wuhan Hubei China; ^6^ Department of Pharmacy Practice, Faculty of Pharmacy University of Tabuk Tabuk Saudi Arabia; ^7^ University Center for Health Science Universidad de Guadalajara Guadalajara Jalisco Mexico; ^8^ Urology and Nephrology Research Center, Shahid Labbafinejad Medical Center Shahid Beheshti University of Medical Sciences Tehran Iran; ^9^ Department of Endocrinology The Fifth Hospital of Wuhan Wuhan Hubei China; ^10^ Department of Rehabilitation Taihe Hospital (Affiliated Hospital of Hubei University of Medical) Shiyan Hubei China

**Keywords:** *Abelmoschus esculentus*, BMI, FBG, HbA1c, okra, type 2 diabetes

## Abstract

Conflicting results have been reported regarding the effects of okra on diabetes biomarkers in patients with type 2 diabetes. These inconsistencies may stem from factors such as dosage, intervention duration, and study design. To address these discrepancies, this systematic review and meta‐analysis of randomized controlled trials (RCTs) was conducted to evaluate the effects of okra ( *Abelmoschus esculentus* ) supplementation on diabetes and obesity markers in patients with type 2 diabetes. Relevant studies published up to July 15, 2024, were identified through comprehensive searches in databases including Scopus, Web of Science, PubMed/Medline, and Embase. Studies were included if they assessed the effects of okra supplementation on diabetes and obesity biomarkers in patients with type 2 diabetes. A random‐effects model was employed to calculate effect sizes, reported as weighted mean differences (WMD) with 95% confidence intervals (CI). A total of six eligible RCTs was included in the final analysis. The meta‐analysis revealed that okra supplementation significantly reduced fasting blood glucose (FBG) (WMD: −21.72 mg/dL, 95% CI: −36.86 to −6.58, *p* = 0.005) and glycated hemoglobin (HbA1c) levels (WMD: −0.42%, 95% CI: −0.64 to −0.19, *p* < 0.001). However, no significant effects were observed on insulin levels (WMD: 0.54 μU/mL, 95% CI: −1.03 to 2.12, *p* = 0.498), insulin resistance as assessed by HOMA‐IR (WMD: −0.31, 95% CI: −1.37 to 0.75, *p* = 0.566), body mass index (BMI) (WMD: −0.24 kg/m^2^, 95% CI: −0.62 to 0.14, *p* = 0.220), or body weight (WMD: −0.83 kg, 95% CI: −2.55 to 0.87, *p* = 0.338). This systematic review and meta‐analysis is the first to confirm the positive effects of okra supplementation on glycemic control in patients with type 2 diabetes, specifically by reducing FBG and HbA1c levels.

**Abbreviations**
BMI: body mass index
BW: body weight
FBG: fasting blood glucose
MeSH: Medical Subject Heading
PRISMA: the Preferred Reporting Items for Systematic Reviews and Meta‐Analyses
RCT: randomized controlled trial
WMD: weighted mean difference

## 1 Introduction

Type 2 diabetes is a major public health concern, contributing significantly to metabolic complications and cardiovascular diseases (CVD) (Ma et al. 2022). It is primarily caused by peripheral insulin resistance and pancreatic beta‐cell dysfunction (American Diabetes Association 2014). Globally, type 2 diabetes accounts for approximately 90% of all diabetes cases, despite widespread preventive measures, leading to high morbidity and mortality rates (Wu et al. 2014). Uncontrolled glucose levels often result in severe diabetes‐related complications, which reduce life expectancy and increase healthcare costs (Deshpande et al. 2008). Therefore, effective blood glucose control is critical for preventing complications in patients with type 2 diabetes (Rakhis Sr. et al. 2022). Despite the availability of various medications for managing type 2 diabetes, the prevalence of diabetes‐related complications and mortality continues to rise (Davies et al. 2022).

Obesity, characterized by excessive accumulation of body fat, is a key risk factor for type 2 diabetes (Yang et al. 2023). A strong correlation exists between type 2 diabetes and body mass index (BMI) (Andréasson et al. 2022). Managing type 2 diabetes often requires weight loss, as these two factors are closely interlinked (Sheng et al. 2024). One proposed mechanism linking obesity and type 2 diabetes is beta‐cell dysfunction induced by excessive adipose tissue and insulin resistance, both of which can be mitigated through weight reduction (Ruze et al. 2023).

Many individuals turn to plants as complementary and alternative therapies to avoid drug‐related complications (Ekor 2014). Plant‐based sources contain bioactive compounds with proven benefits for glycemic control and obesity management (Mao et al. 2021). Among these, okra ( *Abelmoschus esculentus* ), a plant from the Malvaceae family, has shown potential benefits for glycemic control (Durazzo et al. 2018). Okra is rich in minerals, vitamins, antioxidants such as quercetin and flavonoids, dietary fiber, and polysaccharides (Gemede et al. 2018). Diets high in fiber have been demonstrated to improve glycemic control in type 2 diabetes patients (Li et al. 2023). Similarly, flavonoid‐rich diets exhibit antioxidant properties and are effective in preventing obesity and chronic diseases, including diabetes (Abu‐Zaid et al. 2024). Polysaccharides in okra have been shown to reduce glucose levels, promote weight loss, and improve glucose tolerance (Dubey and Mishra 2017). However, conflicting evidence exists, with one study reporting no significant effects of okra on body weight (Nikpayam et al. 2022).

Despite its potential benefits, evidence on the effects of okra on diabetes and obesity markers in humans remains limited. Controversial results regarding okra's impact on diabetes biomarkers in type 2 diabetes patients may stem from factors such as variations in dosage, intervention duration, and study design. To address these inconsistencies, this systematic review and meta‐analysis of randomized controlled trials (RCTs) was conducted to evaluate the effects of okra ( *Abelmoschus esculentus* ) supplementation on diabetes and obesity markers in patients with type 2 diabetes.

## 2 Methods

This systematic review and meta‐analysis aimed to evaluate the effects of okra supplementation on biomarkers associated with diabetes and obesity in individuals with type 2 diabetes. The review was conducted in accordance with the guidelines set forth by the Preferred Reporting Items for Systematic Reviews and Meta‐Analyses (PRISMA) (Moher et al. 2009).

### 2.1 Search Strategy

Relevant studies published up to July 15, 2024, were independently identified by two authors (Y.F. and B.H‐.W.) through comprehensive searches in online databases, including Scopus, Web of Science, PubMed/Medline, and Embase. The search strategy combined both Medical Subject Headings (MeSH) and non‐MeSH keywords, such as (“Okra” OR “ *Abelmoschus esculentus* ”) AND (“Clinical Trials” OR RCT OR “Intervention Studies” OR intervention OR Trial OR “controlled trial” OR randomized OR random OR placebo OR assignment). Additionally, the reference lists of related reviews were manually reviewed to identify any additional relevant studies.

### 2.2 Inclusion Criteria

Studies were included if they met the following criteria: (1) original research using RCT designs; (2) assessed the effects of oral okra supplementation on fasting blood glucose (FBG), insulin levels, homeostasis model assessment of insulin resistance (HOMA‐IR), glycated hemoglobin (HbA1c), BMI, and body weight in patients with type 2 diabetes; and (3) provided the necessary data on these outcomes both at baseline and post‐intervention, or reported the changes in these measures for both the okra and placebo groups.

### 2.3 Exclusion Criteria

Studies were excluded if they met any of the following conditions: (1) in vitro or animal studies; (2) insufficient data at both baseline and post‐treatment; (3) non‐RCT designs; (4) studies assessing the effects of okra in combination with other interventions (co‐supplementation); and (5) unpublished data or gray literature, including dissertations, conference abstracts, and patents.

### 2.4 Data Extraction

Relevant variables from each eligible study were independently extracted by two researchers (L.W. and Y.Z.) according to predetermined criteria. Any disagreements were resolved by a third reviewer. The extracted data included the study location, participants' age and gender, mean and standard deviation (SD) of outcome measures, the first author's name, year of publication, trial duration, participants' health status, okra supplementation dosage, study design, and sample size.

### 2.5 Quality Assessment

The risk of bias in the included studies was independently assessed by two authors (Q.H. and J.M.) using the Cochrane ROB2 tool (Moher et al. 2009; Sterne et al. 2019). This evaluation addressed potential biases related to the randomization process, deviations from the planned interventions, missing outcome data, outcome measurement bias, selective reporting, and overall bias. Based on the assessment criteria, the overall risk of bias for each study was classified as low, some concerns, or high. Additionally, the Grading of Recommendations Assessment, Development, and Evaluation (GRADE) framework was applied to evaluate the certainty of the evidence, which was categorized as very low, low, moderate, or high (Sterne et al. 2019).

### 2.6 Data Synthesis and Statistical Analysis

Treatment responses were assessed by analyzing mean changes and SD of the outcomes from baseline. To combine data and evaluate effect sizes in both the okra and control groups, mean differences with 95% confidence intervals (CI) were calculated. A random‐effects model was used to estimate effect sizes, presented as weighted mean differences (WMD). Standard formulas were applied to compute mean and SD when outcomes were reported in different formats (Higgins and Green 2011; Hozo et al. 2005). In cases where SDs for net changes were not provided, we used the formula: SD_change = √[(SD_pre‐intervention)^2^ + (SD_post‐intervention)^2^ − (2 × *R* × SD_pre‐intervention × SD_post‐intervention)] (Borenstein et al. 2011). Heterogeneity across studies was evaluated using *I* ^2^ statistics, with *p* values below 0.1 and *I* ^2^ values above 50% indicating moderate to high heterogeneity. Sensitivity analysis was conducted to assess the robustness of the pooled effect size by sequentially excluding individual studies. Publication bias was examined using funnel plots, with Begg's and Egger's tests applied to statistically assess asymmetry in the plots (Egger et al. 1997). If publication bias was detected, the “trim and fill” method was used to estimate the potential impact of unpublished studies (Palmer et al. 2008). All statistical analyses were performed using Stata software version 15 (StataCorp, College Station, Texas, USA), with *p* values less than 0.05 considered statistically significant.

## 3 Results

### 3.1 Study Selection

**FIGURE 1:**
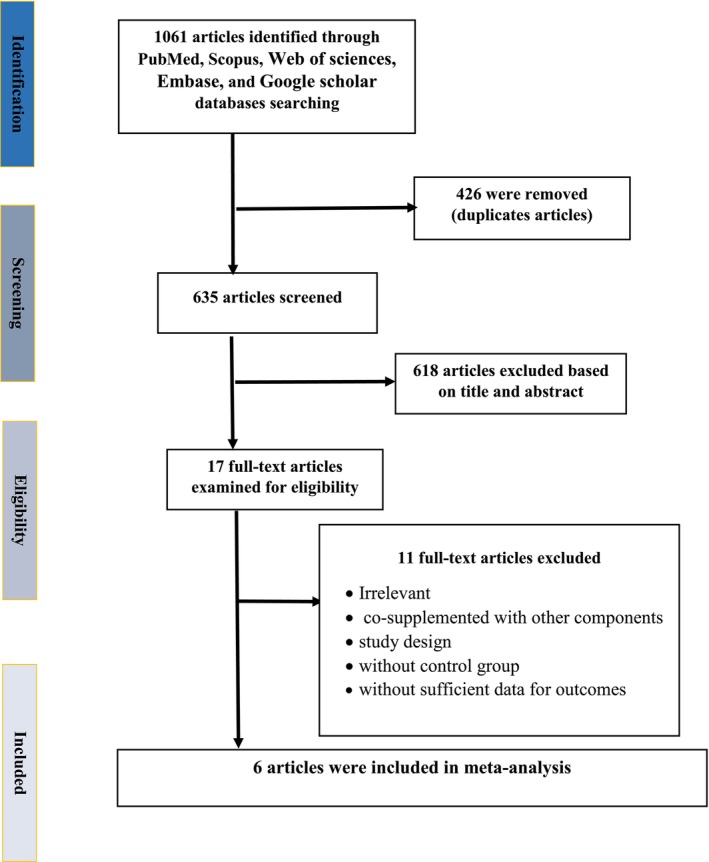
Flow chart for study examined and included into the meta‐analysis.

Figure 1 presents the flow diagram outlining the literature search process. The initial search retrieved 1061 records, of which 426 duplicates were removed. Following the screening of titles and abstracts, 614 articles were excluded. This process resulted in 17 articles being selected for full‐text review, of which 11 were excluded. Ultimately, six eligible studies were included in the final quantitative analysis (Nikpayam et al. 2024; Tavakolizadeh et al. 2023; Chen et al. 2023; Saatchi et al. 2022; Moradi et al. 2020; Haryati et al. 2019).

### 3.2 Characteristics of the Included Studies

Table 1 summarizes the key characteristics of the eligible studies. The duration of okra supplementation in these studies ranged from 2 weeks to 3 months, with daily doses varying from 3 to 20 g. The study populations consisted of individuals diagnosed with type 2 diabetes, and the mean baseline BMI of participants ranged from 24.9 to 30.35 kg/m^2^. These trials, published between 2019 and 2024, were conducted in China, Iran, and Indonesia. The ages of participants in the selected studies ranged from 40.8 to 62 years, and both genders were included.

**TABLE 1:** Characteristics of eligible studies.

Author, year	Country	Population	Participants age (year)	Baseline BMI	Sample size Okra/Placebo	Duration	Okra dosage	Outcomes
Nikpayam et al. (2024)	Iran	Patients with diabetes type 2	62	30.35	30/25	10 weeks	125 mg (4 g of okra)	FBG, insulin, HbA1c, HOMA‐IR, body weight
Tavakolizadeh et al. (2023)	Iran	Patients with diabetes type 2	53.8	28.6	48/46	3 months	3000 powdered okra fruit	FBG, insulin, HbA1c, HOMA‐IR, BMI
Chen et al. (2023)	China	Patients with diabetes type 2	40.8	nr	30/30	60 days	20 g of dried *H. esculentus* fruit tea	FBG, insulin, HbA1c, HOMA‐IR
Saatchi et al. (2022)	Iran	Patients with diabetes type 2	57.7	30.2	50/50	8 weeks	4000 mg/day of okra	FBG, HbA1c, BMI
Moradi et al. (2020)	Iran	Patients with diabetes type 2	54.26	24.9	25/23	8 weeks	10 g okra	FBG, insulin, HbA1c, HOMA‐IR, BMI, body weight
Haryati et al. (2019)	Indonesia	Patients with diabetes type 2	nr	nr	15/15	2 weeks	Three pieces of fresh okra fruit in 250 mL water	FBG

Included RCTs have provided clinical and safety data on okra. Findings indicate that okra significantly reduces FBG and HbA1c without causing adverse effects (Tavakolizadeh et al. 2023). An 8‐week consumption of okra led to a notable decrease in the HOMA‐IR and fasting plasma glucose, with no reported adverse effects (Moradi et al. 2020). Similarly, oral intake of okra whole fruit capsules reduced FBG and HbA1c without any adverse effects (Saatchi et al. 2022). The quality assessment and risk of bias evaluation for the included studies are presented in Table S1. Overall, most studies had a low risk of bias. However, one included study had a risk of bias due to deviations from intended interventions (Haryati et al. 2019).

## 4 Results of Meta‐Analysis

### 4.1 Effects of Okra Supplementation on FBG Levels

**FIGURE 2:**
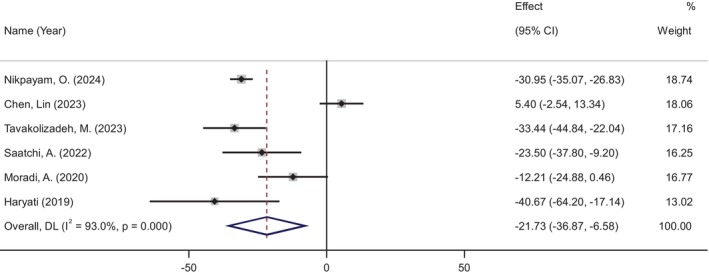
Forest plot of randomized controlled trials investigating the effects of okra supplementation on fasting blood glucose (FBG).

FBG levels were assessed in six studies, which included a total of 387 participants—198 in the okra group and 189 in the placebo group. The meta‐analysis showed a significant reduction in FBG levels following okra supplementation (WMD: −21.72 mg/dL, 95% CI: −36.86 to −6.58, *p* = 0.005), despite significant heterogeneity among the studies (*I* ^2^ = 93%, *p* < 0.001) (Figure 2). Subgroup analyses revealed a more pronounced decrease in FBG levels in RCTs where the daily dose of okra exceeded 4 g (WMD: −30.95 mg/dL, 95% CI: −34.64 to −27.26, *p* < 0.001) and in studies with a duration longer than 2 months (WMD: −31.23 mg/dL, 95% CI: −35.11 to −27.36, *p* < 0.001) (see Figure S1).

### 4.2 Effects of Okra Supplementation on Insulin Levels

**FIGURE 3:**
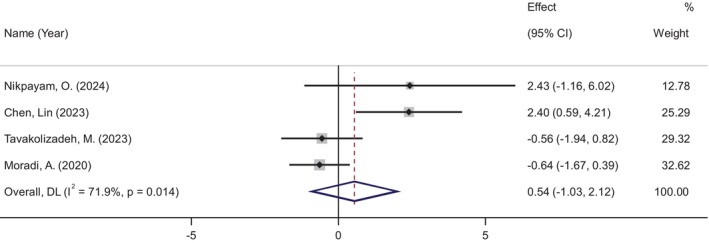
Forest plot of randomized controlled trials investigating the effects of okra supplementation on insulin.

Insulin levels were assessed in four studies involving 257 participants (133 in the okra group and 124 in the placebo group). The meta‐analysis showed no significant change in insulin levels following okra supplementation (WMD: 0.54 μU/ml, 95% CI: −1.03 to 2.12, *p* = 0.498), despite moderate heterogeneity among the studies (*I* ^2^ = 71%, *p* = 0.014) (Figure 3).

### 4.3 Effects of Okra Supplementation on HbA1c Levels

**FIGURE 4:**
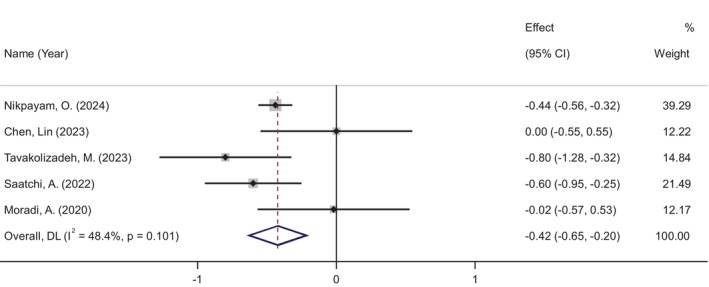
Forest plot of randomized controlled trials investigating the effects of okra supplementation on HbA1c.

HbA1c levels were analyzed in five studies involving 357 participants (183 in the okra group and 174 in the placebo group). The meta‐analysis revealed a significant reduction in HbA1c following okra supplementation (WMD: −0.42%, 95% CI: −0.64 to −0.19, *p* < 0.001), with no significant heterogeneity among the studies (*I* ^2^ = 48%, *p* = 0.101) (Figure 4).

Subgroup analyses showed a more pronounced decrease in HbA1c in RCTs with daily okra doses of ≤ 4 g (WMD: −0.51%, 95% CI: −0.68 to −0.34, *p* < 0.001) and in studies with a duration longer than 2 months (WMD: −0.54%, 95% CI: −0.86 to −0.22, *p* = 0.001) (see Figure S1).

### 4.4 Effects of Okra Supplementation on HOMA‐IR

**FIGURE 5:**
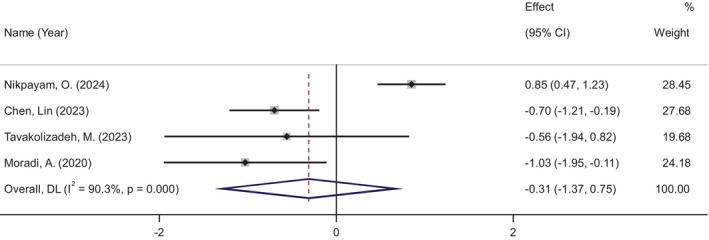
Forest plot of randomized controlled trials investigating the effects of okra supplementation on HOMA‐IR.

HOMA‐IR levels were assessed in four studies involving 257 participants (133 in the okra group and 124 in the placebo group). The meta‐analysis showed no significant change in HOMA‐IR levels following okra supplementation (WMD: −0.31, 95% CI: −1.37 to 0.75, *p* = 0.566), despite high heterogeneity among the studies (*I* ^2^ = 90%, *p* < 0.001) (Figure 5). Subgroup analyses revealed a more pronounced decrease in HOMA‐IR in RCTs with a daily dose of okra greater than 4 g (WMD: −0.77, 95% CI: −1.22 to −0.33, *p* = 0.001) and in studies with a duration of 2 months or less (WMD: −0.77, 95% CI: −1.22 to −0.33, *p* = 0.001) (see Figure S1).

### 4.5 Effects of Okra Supplementation on BMI

**FIGURE 6:**
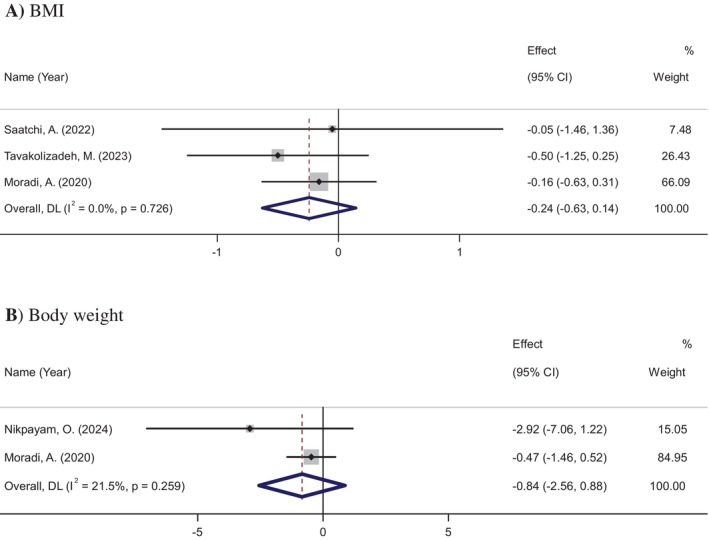
Forest plot of randomized controlled trials investigating the effects of okra supplementation on BMI and body weight.

BMI data were extracted from three studies involving 242 participants (123 in the okra group and 119 in the placebo group). The meta‐analysis showed no significant change in BMI following okra supplementation (WMD: −0.24 kg/m^2^, 95% CI: −0.62 to 0.14, *p* = 0.220), with no significant heterogeneity observed among the studies (*I* ^2^ = 0.0%, *p* = 0.726) (Figure 6A).

### 4.6 Effects of Okra Supplementation on Body Weight

Body weight data were derived from two studies involving 103 participants (55 in the okra group and 48 in the placebo group). The meta‐analysis found no significant change in body weight following okra supplementation (WMD: −0.83 kg, 95% CI: −2.55 to 0.87, *p* = 0.338), with no significant heterogeneity observed among the studies (*I* ^2^ = 21%, *p* = 0.259) (Figure 6B).

### 4.7 Sensitivity Analysis and Publication Bias

**FIGURE 7:**
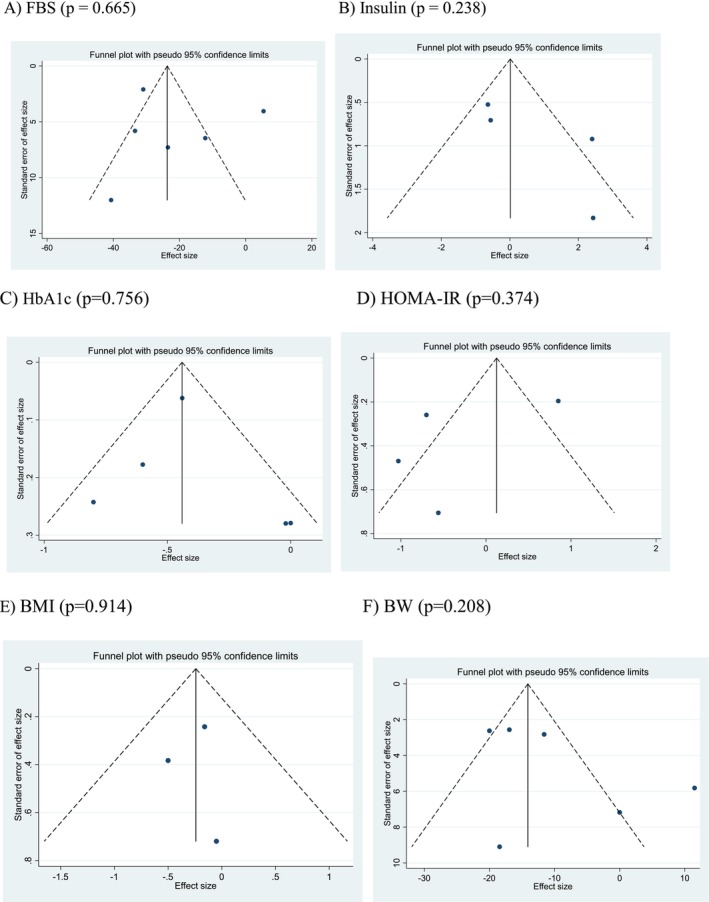
Funnel plot of the weighted mean difference (WMD) versus the s.e. of the weighted mean difference (WMD).

Individual studies did not exert a significant influence on the aggregated effect sizes in the meta‐analysis findings. Both the funnel plot and Egger's linear regression test suggested no presence of publication bias in the meta‐analysis investigating the impact of Okra supplementation on the examined outcomes (Figure 7).

## 5 Discussion

Type 2 diabetes represents a growing public health challenge, with its prevalence rising globally (Hossain et al. 2024). Despite the availability of various pharmaceutical treatments, the incidence of diabetes‐related complications, drug‐associated adverse effects, and mortality remain high (Weinberg Sibony et al. 2023). This underscores the need for effective natural alternatives with fewer side effects. However, unlike pharmaceutical products, nutraceutical manufacturers are not subject to the same stringent regulations regarding the safety, efficacy, and quality of their products, which may lead to the marketing of ineffective supplements (Williamson et al. 2020). Systematic reviews and meta‐analyses offer high‐quality evidence, which is why we conducted this systematic review and meta‐analysis to evaluate the potential benefits of okra, a natural plant, on diabetes and obesity markers in patients with type 2 diabetes. To the best of our knowledge, this is the first systematic review and meta‐analysis based on RCTs to assess the impact of okra supplementation on diabetes and obesity markers in type 2 diabetes patients. After conducting a thorough literature search, we identified six RCTs that investigated the effects of okra in this patient population.

Our analysis found that okra supplementation in type 2 diabetes patients led to significant improvements in diabetes markers, with notable reductions in FBG and HbA1c. The proposed mechanism for okra's ability to lower FBG and HbA1c is its content of both insoluble and soluble fibers. Soluble fiber helps regulate blood glucose by slowing the movement of food through the digestive system, resulting in slower and more gradual sugar absorption. Additionally, dietary fibers increase chyme viscosity, which stimulates gastrointestinal motility and the release of glucagon‐like peptide‐1 (GLP‐1), a hormone responsible for various glucoregulatory effects (Giuntini et al. 2022). However, okra supplementation did not show significant effects on insulin levels or HOMA‐IR concentration. Furthermore, there was no significant impact of okra on obesity indices, including BMI and body weight.

Preclinical studies have shown results consistent with our findings (Wu et al. 2020). One proposed mechanism for okra's ability to reduce FBG levels is its promotion of glycogen synthesis in the liver and the regeneration of pancreatic islets, which enhance insulin secretion and inhibit intestinal glucose absorption, thereby improving glucose uptake (Alblihd et al. 2023). Another proposed mechanism for okra's ability to lower FBG and HbA1c is its content of both insoluble and soluble fibers. Soluble fiber helps regulate blood glucose by slowing the movement of food through the digestive system, resulting in slower and more gradual sugar absorption. Additionally, dietary fibers increase chyme viscosity, which stimulates gastrointestinal motility and the release of GLP‐1, a hormone responsible for various glucoregulatory effects (Giuntini et al. 2022). Additionally, okra may improve glucose homeostasis and beta‐cell function via a peroxisome proliferator‐activated receptor (PPAR)‐dependent pathway (Esmaeilzadeh et al. 2020). PPARs are transcription factors activated by ligands that play a key role in regulating blood glucose.

Oxidative stress, caused by high levels of reactive oxygen species (ROS), is common in type 2 diabetes and is linked to insulin resistance, beta‐cell dysfunction, and impaired glucose tolerance (Dludla et al. 2023). The phenolic compounds in okra help alleviate this stress by reducing ROS, which improves pancreatic beta‐cell function and insulin sensitivity (Sarkar et al. 2022). Okra also contains antioxidants such as vitamins A and C, flavonoids, polyphenols, and quercetin, all of which have been studied for their beneficial effects in type 2 diabetes patients (Saatchi et al. 2022).

Patients with type 2 diabetes are at heightened risk for complications, including reproductive, immune, and cardiovascular disorders (Daryabor et al. 2020). Therefore, effective glucose control is crucial to preventing further complications. Supporting our findings, systematic reviews on animal models have demonstrated that okra has positive effects on glycemic markers such as FBG, HbA1c, and HOMA‐IR, both in acute and chronic treatments. While our meta‐analysis showed no significant change in HOMA‐IR levels following okra supplementation, subgroup analyses revealed a more substantial reduction in HOMA‐IR in RCTs where okra dosage exceeded 4 g/day (WMD: −0.77, 95% CI: −1.22 to −0.33, *p* = 0.001) and in studies with a duration of ≤ 2 months (WMD: −0.77, 95% CI: −1.22 to −0.33, *p* = 0.001).

In our current meta‐analysis, no significant changes in HOMA‐IR and insulin concentrations were observed following okra supplementation. However, Fan et al. (2014) reported a significant improvement in both HOMA‐IR and insulin levels following okra supplementation. The discrepancy in findings may be attributed to the use of animal models in Fan et al.'s study and differences in the dosages of okra supplementation. In our included RCTs, daily doses of okra ranged from 3 to 20 g, while Fan et al. administered 30 g/day in animal models.

Similarly, our meta‐analysis revealed no significant changes in BMI and body weight following okra supplementation. These results are consistent with other studies showing that 10 g of okra powder did not significantly affect BMI or body weight in type 2 diabetes patients (Moradi et al. 2020). Another RCT also demonstrated no effect of okra on body weight in overweight individuals with impaired glucose tolerance (Taniguchi‐Fukatsu et al. 2012). However, some studies reported a significant reduction in body weight following okra supplementation (Jin et al. 2022; Fan et al. 2013). The differences in outcomes may be attributed to the use of animal models in these studies, where animals were fed a high‐fat diet. The polysaccharides in okra have been shown to reduce body weight in such animal models. In humans, the bioavailability of okra may differ due to various factors such as intestinal absorption, gut microbiota, hepatic metabolism, and elimination through bile and urine. These factors could influence the efficacy of okra supplementation.

## 6 Clinical Implications

Okra may serve as an adjunct therapy for controlling blood glucose levels in patients with type 2 diabetes, as it has been shown to reduce FBG and HbA1c. However, it should not be used as a weight loss therapy in these patients. Okra contains fructans, which can cause gastrointestinal disturbances such as diarrhea, bloating, and cramping, particularly in individuals with pre‐existing bowel issues (Fedewa and Rao 2014). While okra can improve blood glucose control, it may interfere with commonly prescribed anti‐diabetic medications, such as metformin, potentially reducing its antihyperglycemic effects (Elkhalifa et al. 2021). Therefore, the potential adverse effects of okra, along with its interactions with food and other medications, should be carefully considered in future studies.

## 7 Strengths and Limitations

This is the first meta‐analysis to investigate the effects of okra supplementation on major diabetes markers and obesity indices in type 2 diabetes patients. The rigorous evaluation of the quality of evidence and risk of bias strengthens the findings of this study. No significant heterogeneity was observed regarding HbA1c.

However, there are several limitations to this meta‐analysis. The review included studies with varying dosages, durations, and formulations of okra, which may explain the observed heterogeneity in some of the outcomes. We conducted sensitivity and subgroup analyses to identify the source of this heterogeneity, though the variability in FBG results may affect the generalizability of our findings. Additionally, the small number of included RCTs (6) and relatively small sample sizes may limit the ability to fully assess the impact of okra on diabetes and obesity biomarkers. As such, the results should be interpreted with caution, as the number of studies examining the effects of okra supplementation in type 2 diabetes patients remains limited. Confounding factors such as diet, exercise, and social habits were not accounted for, which may have influenced the observed relationships between okra supplementation and the outcomes.

## 8 Conclusion

This study is the first systematic review and meta‐analysis to assess the impact of okra supplementation on diabetes markers and obesity indices in type 2 diabetes patients. Our results confirm the favorable impact of okra on diabetes, demonstrated by reductions in FBG and HbA1c following supplementation. Although the available evidence from RCTs is limited, the beneficial effects of okra as an antihyperglycemic agent suggest it could help protect type 2 diabetes patients from further complications. Future research should focus on investigating the adverse effects of okra, its interactions with other foods and medications, and consider additional factors such as diet, social habits, and exercise.

## Author Contributions

**Ying Fan:** conceptualization, formal analysis, methodology. **Lei Wu:** conceptualization, methodology. **Yalan Zhang:** conceptualization, investigation, methodology, writing – original draft. **Qin Hu:** conceptualization, methodology, writing – original draft. **Jing Mei:** methodology, validation. **Kousalya Prabahar:** methodology, writing – original draft, writing – review and editing. **Benjamin Hernández‐Wolters:** data curation, funding acquisition, investigation, methodology, writing – original draft. **Hamed Kord‐Varkaneh:** formal analysis, methodology, writing – original draft. **Changjiang Lei:** conceptualization, supervision, writing – original draft. **Su Zheng:** conceptualization, data curation, methodology, writing – original draft, writing – review and editing.

## Conflicts of Interest

The authors declare no conflicts of interest.

## Supporting information


**Figure S1:** ptr70071‐sup‐0001‐FigureS1.docx.

**Figure S2:** ptr70071‐sup‐0002‐FigureS2.docx.

**Table S1:** ptr70071‐sup‐0003‐TableS1.docx.

## Data Availability

Data are available upon request.
