# Supplementary material for: The Impact of Okra (Abelmoschus esculentus) Supplementation on Diabetes and Obesity Biomarkers in Type 2 Diabetes Patients: A Systematic Review and Meta‐Analysis of Randomized Controlled Trials
Source: Phytother Res. 2025 Aug 27;39(10):4693–703. doi: 10.1002/ptr.70071 (PMC12504793; doi:10.1002/ptr.70071)
Supplement: Supplementary file 1 — Figure S1: ptr70071‐sup‐0001‐FigureS1.docx. [file PTR-39-4693-s003.docx]

**Supplementary Figure 1.** Subgroup analyses

CI, confidence interval.

**1) Length of the intervention (months)**

2) Dose of the Okra(g/day)
