# Supplementary material for: The Impact of Okra (Abelmoschus esculentus) Supplementation on Diabetes and Obesity Biomarkers in Type 2 Diabetes Patients: A Systematic Review and Meta‐Analysis of Randomized Controlled Trials
Source: Phytother Res. 2025 Aug 27;39(10):4693–703. doi: 10.1002/ptr.70071 (PMC12504793; doi:10.1002/ptr.70071)
Supplement: Supplementary file 2 — Figure S2: ptr70071‐sup‐0002‐FigureS2.docx. [file PTR-39-4693-s001.docx]

Supplementary Figure 2.

CI, confidence interval. FBS, fasting blood glucose.

Sensitivity analyses on:

1) FBS

2) Insulin

3) HbA1c

4) HOMA-IR

5)Body mass index (BMI)
