# Supplementary material for: The Impact of Okra (Abelmoschus esculentus) Supplementation on Diabetes and Obesity Biomarkers in Type 2 Diabetes Patients: A Systematic Review and Meta‐Analysis of Randomized Controlled Trials
Source: Phytother Res. 2025 Aug 27;39(10):4693–703. doi: 10.1002/ptr.70071 (PMC12504793; doi:10.1002/ptr.70071)
Supplement: Supplementary file 3 — Table S1: ptr70071‐sup‐0003‐TableS1.docx. [file PTR-39-4693-s002.docx]

| \|  \|  \| \| --- \| --- \| \|  \| 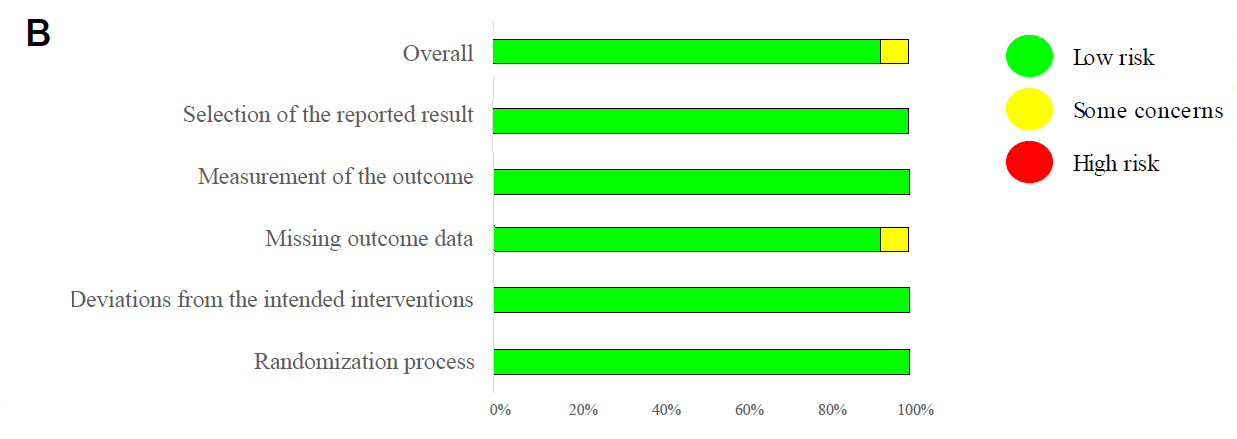 \| \|  \|  \| | Randomization process | Deviations from intended interventions | Missing outcome data | Measurement of the outcome | Selection of the reported result |  |
| --- | --- | --- | --- | --- | --- | --- | --- | --- | --- | --- | --- | --- |
|  | **D1** | **D2** | **D3** | **D4** | **D5** | **Overall** |
| Nikpayam, O, (2024) |  |  |  |  |  | 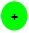 |
| Bahreini, N, (2024) | 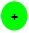 |  | 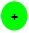 | 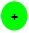 | 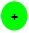 |  |
| Afsharmanesh, Mohammad Reza, (2024) |  |  |  |  |  |  |
| Tavakolizadeh, M, (2023) |  |  |  |  |  |  |
| Chen, Lin, (2023) |  |  |  |  |  |  |
| Saatchi, A, (2022) |  |  |  |  |  |  |
| Moradi, A, (2020) |  |  |  |  |  |  |
| Haryati, (2019) |  | 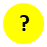 |  |  |  | 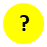 |

Supplementary Table 1: Cochrane risk-of-bias tool for randomized trials version 2 (RoB 2) for each study included in the systematic review assessing the effects of Okra (Abelmoschus esculentus) supplementation on cardiovascular risk factor in typ2 diabetes patients: A meta-analysis of randomized controlled trials.
